# Intraoperative dynamics of workflow disruptions and surgeons' technical performance failures: insights from a simulated operating room

**DOI:** 10.1007/s00464-021-08797-0

**Published:** 2021-11-01

**Authors:** Amelie Koch, Aljoscha Kullmann, Philipp Stefan, Tobias Weinmann, Sebastian F. Baumbach, Marc Lazarovici, Matthias Weigl

**Affiliations:** 1 Institute and Clinic for Occupational, Social and Environmental Medicine, University Hospital, LMU Munich, Munich, Germany; 2 Chair for Computer Aided Medical Procedures & Augmented Reality, Department of Informatics / I 16, Technical University of Munich, Munich, Germany; 3 Department of Orthopaedics and Trauma Surgery, Musculoskeletal University Center Munich (MUM), University Hospital, LMU Munich, Munich, Germany; 4 Institute for Emergency Medicine and Management in Medicine (INM), University Hospital, LMU Munich, Munich, Germany; 5 Institute for Patient Safety, University of Bonn, Bonn, Germany

**Keywords:** Surgical simulation, Flow disruptions, Technical performance, Patient safety

## Abstract

**Introduction:**

Flow disruptions (FD) in the operating room (OR) have been found to adversely affect the levels of stress and cognitive workload of the surgical team. It has been concluded that frequent disruptions also lead to impaired technical performance and subsequently pose a risk to patient safety. However, respective studies are scarce. We therefore aimed to determine if surgical performance failures increase after disruptive events during a complete surgical intervention.

**Methods:**

We set up a mixed-reality-based OR simulation study within a full-team scenario. Eleven orthopaedic surgeons performed a vertebroplasty procedure from incision to closure. Simulations were audio- and videotaped and key surgical instrument movements were automatically tracked to determine performance failures, i.e. injury of critical tissue. Flow disruptions were identified through retrospective video observation and evaluated according to duration, severity, source, and initiation. We applied a multilevel binary logistic regression model to determine the relationship between FDs and technical performance failures. For this purpose, we compared FDs in one-minute intervals before performance failures with intervals without subsequent performance failures.

**Results:**

Average simulation duration was 30:02 min (SD = 10:48 min). In 11 simulated cases, 114 flow disruption events were observed with a mean hourly rate of 20.4 (SD = 5.6) and substantial variation across FD sources. Overall, 53 performance failures were recorded. We observed no relationship between FDs and likelihood of immediate performance failures: Adjusted odds ratio = 1.03 (95% CI 0.46–2.30). Likewise, no evidence could be found for different source types of FDs.

**Conclusion:**

Our study advances previous methodological approaches through the utilisation of a mixed-reality simulation environment, automated surgical performance assessments, and expert-rated observations of FD events. Our data do not support the common assumption that FDs adversely affect technical performance. Yet, future studies should focus on the determining factors, mechanisms, and dynamics underlying our findings.

**Supplementary Information:**

The online version contains supplementary material available at 10.1007/s00464-021-08797-0.

## Introduction

The operating room (OR) remains a challenging workplace for the surgical team [1, 2]. Any additional stress factors potentially increases this risk for patient’s safety and outcomes [3, 4]. One of these challenges are flow disruption events (FDs) which have been found to be ubiquitous in the OR [5, 6]. FD incidents have been described as 'deviations of the natural progression of the operative procedure' [7, 8]. Phone calls, visiting external staff members, or defective medical equipment—there is a long list of potentially notable FD events [9, 10]. They have been found to be highly prevalent with reported occurrence rates of up to 13 FDs per hour [11, 12].

FDs pose pressure and strains to the OR team on top of the inevitable demands of complex surgical interventions [13]. Team members may be less able to adapt to complex and high-risk situations when coping with additional demands triggered through FDs [14]. The impact range and frequencies of intraoperative FDs have been investigated previously with inconsistent results [15]. At the same time, there is often a lack of a deeper view on the duration, source, and degree of severity of FDs. It has been emphasised that the focus should be on those FDs which are most severe and at the same time most prevalent [14].

Several studies found that FD events adversely affect the levels of stress, cognitive workload, and perceived distraction of the surgical team [12, 16–18]. Even though those findings may lead to the preliminary conclusion that FDs negatively affect surgical performance, the current study base does not provide sufficient evidence for this claim [19]. Some considerations challenge this conclusion. First, system factors, component factors, as well as protective team mechanisms, might counteract or even compensate for increased levels of stress due to FDs. Second, the level of expertise and practice routine of a surgical team can moderate the impact of FDs [20]. It can be hypothesised that these factors counteract possible deteriorations in surgical performance due to FDs.

Studies investigating the impact of FDs on performance failures are rare. Simulation-based settings have been found to be suitable for conducting randomised and controlled studies on relationships and effects that cannot be investigated in the real OR due to ethical considerations [21, 22]. In real-world surgical settings, ethical concerns might be raised when FDs are artificially or deliberatively created with unforeseen risks for patient safety. Moreover, simulations offer unique opportunities for automated, objective technical performance measurements [23, 24]. Technical skill indicators that are most frequently measured are time to task/surgery completion, economy of motion, tool movement smoothness, instrument path length, errors, and final quality [25]. However, current simulations tend to oversimplify the actual OR situations and are, therefore, limited in their external validity [26].

We set out to combine the methodological advantages of simulation settings with those of video-based observations: the possibility of objectively quantifying surgeons’ technical performance [27] and transferability to surgical practice [28]. Additionally, retrospective video observations allow discerning the chronological order of FDs and performance enabling inferences concerning causal evidence.

Following these thoughts, our aim was to determine whether the probability of surgical performance failures increases as a direct effect of FD events. Specifically, we focussed on the following hypotheses:

1. There is no immediate effect of FDs on performance failures (e.g. injuries of the spinal cord).
2. There are specific types of sources of FDs that cause performance failures at a higher rate.
3. The duration and severity of FD events do make a significant difference regarding the association between FDs and performance failures.

## Materials and methods

### Study design and participant recruitment

This is a mixed-reality-based OR simulation study. Ethical Approval was provided by the Ethics Committee of the Faculty of Medicine of the LMU University (No 773-15) before the start of the study. Surgeons were recruited through snowball invitation from four surgical departments performing vertebroplasties of two local university hospitals in Munich, Germany. Written consent for study participation and data assessment was obtained before the study. We followed the reporting guidelines for observational studies (STROBE) and the extensions for simulation studies in health care [29].

### OR setting and surgical procedure

We set up a full-scale surgical team simulation with a 3D-printed patient anatomy model, simulated X-ray imaging, and a fully equipped OR set-up. Participants performed a simulated vertebroplasty (VP) procedure from incision to closure whilst being accompanied by a complete, confederate surgical team (i.e. scrub nurse and anaesthetist). Furthermore, the circulating nurse (also a member of the study team) operated the C-arm. The simulation was conducted in a fully equipped operating room in a simulation centre. In addition to the OR team, one observer was present in the OR.

A 3D-printed spine model with synthetic soft tissue enabled a realistic performance of all required steps for a two-sided VP of a fractured lumbar vertebra. The spine model was based on real patient computed tomography (CT) data. Percutaneous VP is a frequently performed intervention for osteoporotic vertebrae fractures. The aim is to inject bone cement through the pedicle into the fractured vertebrae for stabilisation and pain relief. Percutaneous VP comprises of the following three essential steps: (1) Identifying the ideal entry point on the skin, (2) insertion of the trocar into the pedicle and advancement into the vertebral body, (3) injection of bone cement [30]. A radiation-free C-arm and monitor with realistically simulated X-ray images and all necessary surgical equipment (i.e. scalpel, hammer, cement) were provided. The surgical simulation environment had already been successfully piloted [31]. Technical aspects of the simulated setup are described in detail elsewhere [31].

### Procedure simulation and data collection procedure

Participating surgeons were first given the opportunity to familiarise themselves briefly with the patient case and plan the intervention on a workstation outside the simulated OR. After entering the simulated OR and introducing themselves to the acting surgical team, participants familiarised themselves with the simulation set-up. Subsequently, they conducted a complete vertebroplasty from incision to suture. After completion of the simulation task, participants were asked to fill in a questionnaire on demographic details and the realism and fidelity of the simulation setup.

Each simulation was audio- and videotaped by two opposite cameras from incision to closure. The observation and assessment of occurring flow disruptions were retrospectively carried out by two trained expert raters (authors AK & MW). Both raters have expertise in teamwork assessments in surgical teams, completed pilot observations during real-world surgeries, and have experience with similar simulation studies [32]. Both were blinded to participants’ identities. The data assessment periods were defined by the surgeon and began when they stated that they would start and ended when the surgeon explicitly stated they finished. FDs were identified through video review, classified, and entered in a separate datasheet. To ensure the quality of FD identification, two videos were coded by two expert raters, and interrater reliability (IRR) was determined. Questions and inconsistencies were resolved through discussion. Technical performance data were automatically measured utilising instrument tracking data and anatomically labelled CT data (see below ‘Intraoperative technical performance’).

### Measurements and data sources

#### Flow disruptions

Each observed FD event was evaluated according to its time of occurrence, duration, severity, and source. Additionally, we recorded if the FD event was self-initiated by the participating surgeon (e.g. a question regarding the study process). Examples for each source category can be found in Table 1. We used a slightly modified version of an observation tool for intraoperative flow disruptions that has been used before [33–35].

**Table 1:** Observation tool for surgical flow disruption in simulated ORs

FD category	Definition	Examples
External factors	External cause that has nothing to do with the surgical case or the ongoing surgery	– Small talk, case-irrelevant communication – External calls, ringing phones – Door openings, disruptive visitors
Communication	Verbal and non-verbal communication failures	– Statements are either not or poorly understood and must be repeated
Equipment	Equipment failures or breakdowns	– Cement injection does not work – Simulated C-arm X-ray imaging not working – Malfunctioning of [simulation] medical equipment
Coordination	Staff errors or failures, failure of task coordination	– Nurse fails to properly prepare the cement in time – Handling of the C-arm delays further proceeding – Required medical instrument has not been prepared
Surgeon task considerations	Reconsideration of the next procedure steps	– Difficulties finding the correct access paths into the vertebrae – Speaks up on how to proceed further
Environmental factors	Adverse environmental conditions or changes	– Light changes – Changes in room temperature – Alarms, sounds from medical devices
Simulation	Uncertainties related to the simulation, issues with the handling of the simulator	– Questions about the handling of specific parts of the simulation – Self-initiated interactions with the study team

Additionally, each FD was rated concerning its severity (see Table 2). This rating is based on similar assessments that determined the level of interference or involvement within an interrupting event [36, 37].

**Table 2:** Severity rating of flow disruption events

Rating:	0	1	2
	Potential Distraction	Multi-tasking	Interruption (Break in task activity)
Definition	Events happening in the background that may distract surgeons attention	Events engaging surgeon in a second task simultaneously	Events causing an actual break in surgical primary task activity
Examples	– Background equipment alarms – Unanswered Phone calls – Door openings/visitors without interaction	– Small talk with the team – Questions about simulation (without break in simulation task)^a^ – Communication problems (i.e. misunderstandings)	– Restarting the simulator software^a^ – Breaks to think about next step

^a^Events that occur exclusively in the simulated environment and that do not take place in the real OR

#### Intraoperative technical performance

Intraoperative tracking data of the primary surgical instruments, i.e. the trocar and anatomically labelled CT data were used to determine surgical performance failures. Performance assessment based on instrument tracking has been shown to be highly objective with strong evidence for validity of scores [24]. We defined performance failures as any injury to critical tissue or bone structures (e.g. spinal cord). To correct for possible minor imprecisions of instrument tracking, all incidents were excluded if the instrument remained in a critical structure for less than 5 s.

#### Potential confounding influences

Since it has been shown that surgical expertise may influence the impact of FDs [14, 20], expertise measured as numbers of previously performed VPs and previous experience with surgical simulations (yes/no) were obtained.

### Data extraction

**Fig. 1:**
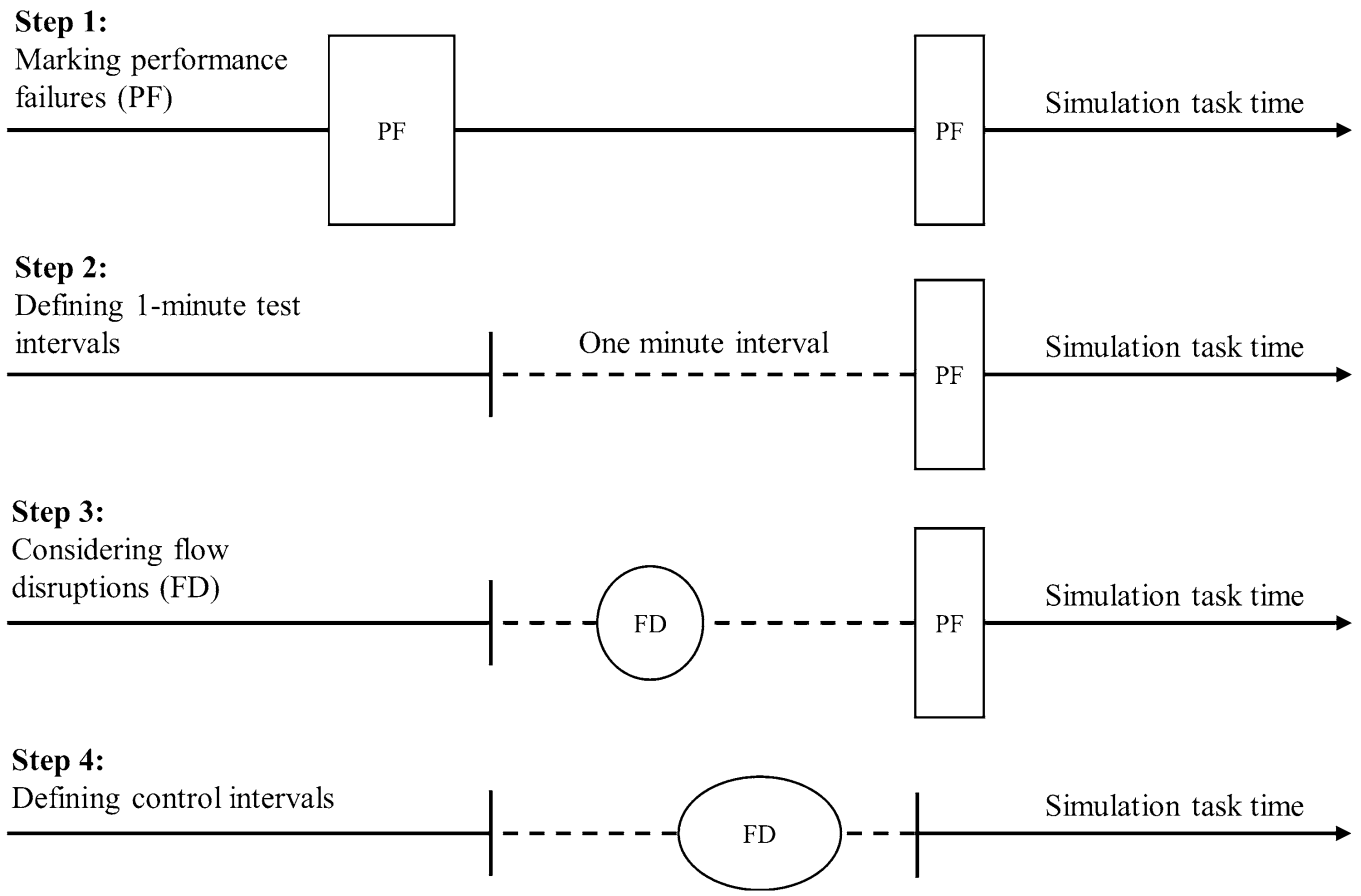
Illustration of data preparation process

To draw conclusions about the prospective relationship between the occurrence of FDs and potentially consequent performance failures, an analysis approach was established that accounted for the incidence (i.e. test vs. control periods) and chronological sequence of FD and performance outcomes (cf., Fig. 1).

In the first step, we identified all performance failure events and, secondly, determined one-minute time periods prior to each event. Thirdly, we counted FDs that occurred within this one-minute time periods prior to failures. We chose this duration based on previous research observing that most surgical FDs are handled within 1 min [38, 39]. In the fourth step, we randomly selected time periods without any performance failure for each operation, respectively, (i.e. control intervals) and counted occurred FDs. In summary, we thus established an analysis approach for the likelihood of FDs that matched time intervals immediately before performance failures with a respective number of intervals without any failures. As two participants did not commit any failures, an average number of control intervals per participant was added in these two cases.

### Statistical analyses

Analyses comprised descriptive statistics for all outcome measures. Subsequently, we applied a multilevel binary logistic regression model to explore the relationship between FDs and technical performance. This modelling approach allows accounting for clustered data structures, i.e. clustered FD events within one procedure. The following analyses were deployed:

1. Main analyses: The extent of the relationship between occurrence of FDs (yes/no) and performance failures was quantified using odds ratio. Surgical expertise was included as a potential confounder.
2. Sensitivity analyses: We checked whether the odds ratio would change after replacing the binary predictor FDs (yes/no) with the actual count of FDs for each one-minute interval, i.e. since multiple FDs can occur within a one-minute interval.
3. FD categories: We assessed how the different categories of FDs were individually associated with performance failures.
4. FD severity and duration: We tested whether the relationship between FDs and performance failures would change when the duration or severity of the respective FD was used in the model instead of the binary predictor of FDs (yes/no).

All data have been entered and further processed in SPSS Statistics (IBM) with the Generalized Linear Mixed Models (GLM) function. We set a *p*-level of 0.05 for all statistical analyses. To address the risk of multiple testing, we applied a Bonferroni correction for all multivariate analyses: corrected *p*-level 0.05/8 = 0.00625.

## Results

### Participants

Eleven surgeons participated in the study. Eight participants were male (72.7%) and were working in either trauma or orthopaedic surgical departments. Mean working experience was 7.8 years (range 0 to 33 years). Previously performed vertebroplasties ranged from 0 to 200 with a mean (M) of 35.0 and a standard deviation (SD) of 62.8. Five surgeons (45.5%) reported previous experiences with surgical simulations. Each surgeon was accompanied by a mock-up OR team (scrub nurse and anaesthesiologist). Procedure duration ranged from 11:15 to 47:05 min (*M* = 30:02 min; SD = 10:48).

### Flow disruptions: rates, distribution, and characterisations

Overall, 114 flow disruption events were observed. Table 3 lists rates of FDs per hour, the proportion of self-initiated events, severity ratings, and event duration. The mean hourly rate of all FDs was 20.4 (SD = 5.6) with substantial variations across FD sources. On average, 34.2 FDs (SD = 12.26) were self-initiated by the surgeons with 70.1% in individual FD categories, i.e. related to the simulation procedure. The median severity rating of all FDs was 1. The average duration of FDs ranged from 3.5 to 23.7 s with an overall mean of 13.2 s (SD = 14.5).

**Table 3:** Rate and distribution of observed flow disruptions

FD source (category)	FDs (count)	FD rate (per h)	Self-initiated FDs (in %)	FD duration (in s)	FD severity (scale 0–2)
		Mean (SD)			Median
Simulation-related	71	12.3 (5.0)	70.1 (20.7)	09.0 (09.7)	1
External factors	29	5.0 (4.7)	34.9 (40.5)	23.7 (27.8)	0
Coordination	6	1.0 (1.5)	0 (0)	17.5 (18.5)	2
Communication	4	0.6 (1.0)	0 (0)	3.5 (0.6)	1
Equipment	3	0.5 (0.9)	0 (0)	18.3 (15.7)	2
Surgeon task considerations	1	0.1 (0.4)	100 (0)	7.0 (0)	2
Total	114	20.4 (5.2)	34.2 (12.3)	13.2 (14.5)	1

*SD* standard deviations; sorted by total count; n = 11 participants; FD severity: 0 = distraction, 1 = multi-tasking, 2 = interruption/break in task activity

### Technical performance outcomes

Of all 11 surgeons, two completed the task without any performance failure such as harming or injuring critical structures. Observed counts for each different type of technical performance failures are listed in Table 4.

**Table 4:** Descriptive statistics of performance failures during simulated vertebroplasties

Performance failures	Count (%)	Rate (per h)	Duration (in s)^a^
		Mean (SD)	
Vertebra perforation	20 (37.7)	3.4 (7.7)	13.4 (10.1)
Pedicle perforation	15 (28.3)	2.4 (2.1)	13.4 (9.9)
Injury facet joint	7 (13.2)	1.0 (1.8)	7.9 (4.0)
Injury spinal cord	7 (13.2)	1.1 (1.6)	13.7 (17.3)
Injury of liver tissue	2 (3.8)	0.4 (1.0)	21.4 (22.5)
Injury of intervertebral disc	1 (1.9)	0.2 (0.6)	6.7 (0)
Injury of kidney tissue	1 (1.9)	0.2 (0.6)	10.9 (0)
Total	53 (100.0)	8.6 (3.3)	12.5 (10.8)

^a^Time period when the instrument was tracked within the critical area; *n* = 11 participants

Altogether, 53 performance failures were recorded with unintended vertebra perforations being most frequent (37.7%). This corresponds to the mean hourly rate of technical performance events of 8.6 (SD = 3.3) with a range from 3.4 (vertebra perforation) to 0.2 (injuries of intervertebral disc or kidney tissue). The mean duration of all performance failures was 12.5 s (SD = 10.8).

### Analyses for associations between FDs and performance failures

A total amount of 118 intervals were included in our analyses with 37 intervals of one or more FDs. Descriptive statistics used for the multivariate analyses are depicted in the digital supplementary files (see Supplementary File 1). As described above, the mixed-effects modelling consisted of four consecutive steps.

First, a logistic regression model was established that tested for effects of FDs (FD yes/no) on the performance outcome, i.e. performance failure (yes/no). We controlled for surgical expertise (number of previously performed VPs, experience with surgical simulations). This model showed an adjusted odds ratio of 1.03 (95% CI 0.46–2.30) indicating no statistically significant relationship between FDs and performance failures (Table 5**).**

**Table 5:** Multilevel logistic regression analyses of individual FDs categories and technical failures

	*n* intervals (*n* intervals with FDs)	Adjusted odds ratio^a^ (95% CI)		*p* value
FDs yes/no	118 (37)	1.03 (0.46–2.30)		0.94
Simulation-related FDs	108 (27)	1.32 (0.53–3.29)		0.55
External FDs	87 (6)	0.27 (0.03–2.55)		0.25
Self-initiated FDs				
Yes	100 (19)	1.07 (0.38–2.98)		0.90
No	99 (18)	0.94 (0.32–2.71)		0.90

^a^Dependent variable: performance failure (yes/no); Confounder: # of vertebroplasties performed and previous experience with surgical simulators (yes/no)

Secondly, our sensitivity analysis revealed that this odds ratio measure did not change substantially after the binary predictor FDs (yes/no) was replaced by the actual number of FDs: odds ratio = 0.92 (95% CI 0.51–1.66). Consequently, in cases where multiple FDs were recorded, we then merely included in the following analyses the most recent FD prior to the performance failure within intervals.

Thirdly, we then calculated regression estimates for the two most frequent FD source categories. None of the individual FD sources was statistically significantly related to performance failures. Moreover, we assessed if the relationship of FDs and performance failures was different depending on whether the FDs are self-initiated or not. In both groups, FDs remained unrelated to performance failures (Table 5).

In our fourth and final step, we replaced the binary independent variable (FDs yes/no) with the FD duration or FD severity ratings as predictors. Again, no statistically significant associations between FDs and performance failures were observed. More details of the additional analyses can be found in Supplementary File 2.

Finally, we assessed the relationship between occurring FDs (count per procedure) and “time to resolve the surgical task” (procedure duration) as an additional performance outcome and found a strong correlation of *r* = 0.820.

## Discussion

Surgical errors can have a devastating effect on the surgical outcome [40]. The evidence base remains inconsistent concerning the ramifications of FDs on surgical performance and outcomes [15, 41]. Notwithstanding, precarious sources of errors and patient harm associated with FD need to be mitigated [13, 15, 42, 43]. We, therefore, set out a study assessing the effects of FDs on technical performance failures. Our simulation approach is without ethical concerns (i.e. no patient harm) with yet precise objective intraoperative performance measurements and behaviour observation during surgical workload.

According to our data, we identified no relationship between FDs and the likelihood of immediately following performance failures. No evidence was also found that the different source types of FDs affect this relationship. Furthermore, our data revealed that the duration or the severity degree of the observed FDs had no impact on this association.

The fact that our study did not provide evidence for an association between FD events and the performance failures under investigation in a simulated vertebroplasty procedure task is, to some extent, consistent with previous findings [9, 44]: Sujka et al. reported no effect of pager interruptions on performance in a simulated laparoscopic cholecystectomy trial [45]. Goodell et al. found no significant effects of cognitive distraction on error rates [46]. Weigl et al. found a significant increase in surgeons' workload in a disrupted scenario yet no effect of FDs on surgical performance [30].

Post hoc, the following explanations are conceivable for our findings: FDs induced by the surgical team, such as small talk or teaching, may be initiated at opportune moments. The surgical team might thus either be resilient to the negative effects of FDs [47] or there are compensatory mechanisms that hinder FDs from having a direct negative effect on technical performance, e.g. implicit safety nets. Furthermore, in cognitive science, it has been shown that workflow disruptions negatively affect mental functions such as attention span and memory performance [48]. However, our findings indicate that in hands-on practice settings technical performance is not necessarily adversely affected by FDs.

In the surgical literature, some studies also identified adverse effects due to interruption events [26, 49, 50]. This is contrasting to the above mentioned findings and might be attributed to various reasons. First, it has been proposed that the nature of FD events (i.e. source types) may modify their effects on performance [30]. Secondly, the level of difficulty and complexity of the surgical task, surgeon’s expertise, the familiarity of the surgical team, the duration, as well as the severity of FD events have been suggested as modulating influences [5, 14, 51]. We thus controlled for the surgeons’ experience and specified the degree of severity, duration, initiation, and source of the observed FDs. Still, these had no significant association with performance failures.

Our full-scale and controlled OR simulation provides a unique opportunity to investigate the effects of FDs during surgical interventions. It resembles a controlled environment whilst rendering the natural characteristics of an OR setting. Contrary to previous field studies, these simulations allow for objective and quantitative measurements of technical surgical performance. Although previous observational studies found very little effect of FDs on surgical performance, we aimed to investigate whether this might change in micro-level observations within brief time intervals, i.e. 1-min episodes. Preceding studies used FDs intentionally induced (or manipulated) by the investigators. But deliberatively triggered FDs might not reflect the true nature of actual FD events in the OR [26]. Moreover, such investigations may neglect FDs initialised by the OR team itself, such as small talk or coordinative discussions. In our study self-initiated FDs accounted for 34.2% of all FDs. Therefore, the herein chosen full-scaled and controlled OR simulation increases the transferability to real surgical practice. Moreover, our thorough characterisation of intraoperative FDs through retrospective video observations is a major advantage of this study compared to previous observational studies in the OR. Nonetheless, observations in naturalistic OR settings have genuine value for external validity and insights into ‘work-as-done’.

A final issue, being repeatedly discussed in literature, is causality in the relationship of FDs and adjacent performance outcomes that cannot be addressed through correlational designs [17, 52]. Our episodic and fine-grained approach involves the comparisons of one-minute intervals prior to performance failure events with intervals without any subsequent performance failures. Our approach thus accounts for the chronological order of FDs and consequential performance failures.

### Limitations

Two major issues limit the validity of our findings: First, the most important limitation of this study is the small number of participants. But, the herein included number of surgeons is similar to most previous studies [45, 46, 50, 53]. Since the realisation of such full-scale team simulations is very time-consuming and surgeons hardly find availabilities alongside their intense hospital routines to participate, it is often difficult to recruit a large number of study attendees. Still, our findings need to be replicated based on larger samples. To increase the transferability of the results to longer lasting surgeries, task times should also be extended. Secondly, a common issue with surgical simulation studies is that the complexity, strains, and pressure of a real OR cannot be adequately reproduced. It is possible that a reduced feeling of personal responsibility for the case or a lower degree of pressure to succeed may have led to more errors being made than in real ORs [26]. Unfortunately, we are not able to determine the exact amount of this bias. As part of further data evaluation, we asked surgeons to rate the realism of the simulation on a Likert scale from 1 (“does not apply at all”) to 5 (“completely applies”). The average score was high with 4.5 (SD = 0.5) confirming a high perception of realism amongst participants. The transferability of our findings to daily surgical practice needs to be considered carefully for another reason: our study applies a micro-level approach and focuses on small time intervals. It is conceivable that the contribution of contextual factors (i.e. ergonomic conditions, resource constraints) to the relationship of FDs and surgical performance is underestimated.

Additionally, few minor limitations should be considered. First, some consequences of frequent FDs on performance cannot be detected by our study design: this includes long-term effects of FDs on surgeon’s decision making, mental resources, and fatigue, as well as accumulated effects of consecutive FDs (i.e. FD cascades) [12, 54]. It has been suspected that only severe or accumulated FDs may have a negative effect on surgical performance [55]. Secondly, we have restricted this study to technical performances with a particular focus on accuracy and maintaining safety margins to critical anatomy, tissue, or structures. Further technical skills such as economy of motion or speed as well as non-technical skills were not taken into account. In addition, we defined any perforation of critical structures as a performance failure event. The various degrees of severity of these injuries to sensitive structures were not taken into account. Thirdly, we investigated a specific task simulation in spine surgery. The transferability of these findings to other surgical specialities needs to be considered carefully. Fourthly, we included FDs in our analysis that would not be expected in the real OR (i.e. questions about the simulation process). It is also feasible that FDs that commonly occur in real-world ORs have been neglected here (i.e. frequent visitors or change of staff). Furthermore, due to the various existing definitions of FDs, it is conceivable that we did not include all potentially relevant FDs. Finally, the role of surgeons’ expertise across different stages of speciality training as well individual coping skills in dealing with intraoperative FDs warrant further investigations.

### Implications

Surgical practice takes place in complex socio-technical environments. Future studies should focus on determining factors, mechanisms, and dynamics shaping the relationship between FDs and performance. Contextual factors, such as ergonomic conditions, need to be given greater attention as well. Simulation studies are a viable option for investigating FDs, particularly within mixed-reality designs that mimic the reality of an OR to a high degree. Since many simulation studies involved too few participants, many contributing factors (i.e. surgical expertise, sources of FDs) and their interaction have not yet been sufficiently taken into account. In particular, larger samples with varying contextual factors and multiple types of surgeries are necessary. Future investigations should also identify those FDs that actually have the potential to result in harm to the patient in the course of postoperative recovery, i.e. mortality and morbidity outcomes. This is especially important to finally be able to provide clear recommendations for the practical handling of FD events in surgical and clinical practice.

Surgical teams are permanently exposed to FDs such as pager calls, student questions, or missing equipment as well as disrupt each other with small talks or coordinative failures [10]. We deem that non-disruptive OR environments support provider performance and patient safety. Similar findings have been made in other sectors such as aviation (i.e. sterile cockpit) and driving [56, 57]. But since this is neither feasible nor always the best solution for clinical day-to-day practice, further efforts should be made to identify those FD events that are detrimental to procedure flow and cause harm. Ultimately, the goal is to eliminate avoidable and harmful FDs, minimise adverse effects of necessary FDs, and allow for or foster those FDs with positive effects for provider cognition, teamwork, and surgical care.

## Conclusion

Our study did not provide evidence for an association between FDs and performance failures. Through the utilisation of a mixed-reality and team simulation environment, automated surgical performance assessments, and retrospective observational analyses of video records, our study extends previous methodological approaches into FD-performance relationships. To further advance the current evidence base, we recommend future studies to take a holistic view and focus on the mechanisms in the relationship between surgical flow disruptions and patient outcomes and safety.

## Supplementary Information

Below is the link to the electronic supplementary material.

Supplementary file1 (DOCX 20 KB)
