# Supplementary material for: Intraoperative dynamics of workflow disruptions and surgeons' technical performance failures: insights from a simulated operating room
Source: Surg Endosc. 2021 Nov 1;36(6):4452–61. doi: 10.1007/s00464-021-08797-0 (PMC9085674; doi:10.1007/s00464-021-08797-0)
Supplement: Supplementary file 1 — Supplementary file1 (DOCX 20 KB) [file 464_2021_8797_MOESM1_ESM.docx]

# Supplementary Digital file 1

**Table 1. Descriptive statistics of FDs included in multivariate analyses of one minute intervals (n=118)**

| **FD source**  (category) | **FDs**  (count) | **Self-initiated FDs**  (in %) | **FD duration**  (in sec) |  | **FD severity**  (scale 0-2) |
| --- | --- | --- | --- | --- | --- |
|  |  |  | **Mean (SD)** |  | **Median** |
| Simulation-related | 27 | 63.0 | 10.0 (9.2) |  | 1 |
| External Factors | 6 | 33.3 | 36.8 (44.9) |  | 0 |
| Coordination | 2 | 0.0 | 30.5 (33.2) |  | 1/2 |
| Communication | 2 | 0.0 | 4.0 (0.0) |  | 1 |

Note: SD Standard deviations; sorted by total count; n=118 intervals; FD severity: 0=distraction, 1=multi-tasking, 2=interruption/break in task activity; FDs with category ‘equipment’ and ‘surgeon task considerations’ did not occur during included one minute intervals

# Supplementary Digital file 2:

# Additional analyses for FD duration, severity and a combined risk score (duration*severity)

**Table 2. Multilevel logistic regression analyses of FDs and technical failures with different coding of FD events**

| **Coding of FDs (predictor)** | **n intervals**  (n intervals with FDs) | **Adjusted odds ratio^1^**  (95% CI) | **p value** |
| --- | --- | --- | --- |
| FDs yes/no | 118 (37) | 1.03 (0.46-2.30) | 0.94 |
| FDs duration | 118 (37) | 0.93 (0.85-1.02) | 0.11 |
| FDs severity | 118 (37) | 1.87 (0.45-7.85) | 0.38 |
| FDs risk score (duration*severity) | 118 (37) | 0.97 (0.93-1.01) | 0.10 |

NOTE: ^1^Dependent variable: performance failure (yes/no); Confounder: # of vertebroplasties performed and previous experience with surgical simulators (yes/no)
